# Investigating the early-life determinants of illness in Africa: the Drakenstein Child Health Study

**DOI:** 10.1136/thoraxjnl-2014-206242

**Published:** 2014-09-16

**Authors:** H J Zar, W Barnett, L Myer, D J Stein, M P Nicol

**Affiliations:** 1 Department of Paediatrics and Child Health, Red Cross War Memorial Children's Hospital, University of Cape Town, Cape Town, South Africa; 2 Division of Epidemiology and Biostatistics, School of Public Health & Family Medicine, University of Cape Town, Cape Town, South Africa; 3 Department of Psychiatry and Mental Health and MRC Unit on Anxiety & Stress Disorders, University of Cape Town, Cape Town, South Africa; 4 Department of Clinical Laboratory Sciences, University of Cape Town, Cape Town, South Africa

**Keywords:** Pneumonia

## Abstract

Respiratory disease is the predominant cause of illness in children globally. We describe a unique multidisciplinary South African birth cohort, the Drakenstein Child Health Study (DCHS), to investigate the incidence, risk factors, aetiology and long-term impact of early lower respiratory tract infection (LRTI) on child health. Pregnant women from a poor, peri-urban community with high exposure to infectious diseases and environmental risk factors are enrolled with 1000 mother–child pairs followed for at least 5 years. Biomedical, environmental, psychosocial and demographic risk factors are longitudinally measured. Environmental exposures are measured using monitors placed at home visits. Lung function is measured in children at 6 weeks, annually and during LRTI episodes. Microbiological investigations including microbiome and multiplex PCR measures are done longitudinally and at LRTI episodes. The DCHS is a unique African birth cohort study that uses sophisticated measures to comprehensively investigate the early-life determinants of child health in an impoverished area of the world.

## Background

Globally, childhood respiratory illness is a major cause of morbidity and mortality.^1^ Childhood pneumonia or lower respiratory tract infection (LRTI) remains the main cause of mortality, while asthma is the most common non-communicable disease. The burden of childhood respiratory illness is highest in low and middle income countries (LMICs), reflecting the demographic profile, epidemiology of LRTIs, distribution of risk factors and weak health systems in many settings.^2^ Several risk factors for LRTI have been identified, but there are limited data on the interaction or cumulative effects of these on incidence and long-term health. Further, there are few data from LMICs on the impact of immunisation with conjugate vaccines against *Streptococcus pneumoniae* and *Haemophilus influenzae* b on the burden and aetiology of LRTI.^3^

Several birth cohort studies in high-income countries have investigated the determinants of chronic respiratory illness, including wheezing illness or asthma.^4^ These suggest that LRTI early in life may result in chronic illness. A meta-analysis reported a 5%–14% risk of developing a major respiratory sequelae following childhood LRTI.^5^ Malnutrition, poverty or crowded living conditions increase this risk. Other factors such as allergen, biomass or bacterial exposure may also lead to chronic disease dependent on the intensity, duration or timing of exposure and genetic susceptibility. Although the burden of LRTI is especially high in African children, there have been no similar cohort studies conducted in this setting.

The Drakenstein Child Health Study (DCHS) aims to investigate the epidemiology, risk factors, aetiology and long-term impact of early LRTI on child health in an LMIC. This study is unique given its location in a poor African community and the ability to undertake sophisticated measures of disease and of risk factors longitudinally from the antenatal period through early childhood. The focus on LRTI addresses a global priority in child health, for which no similar birth cohort studies have been undertaken.

## Methods

**Figure 1:**
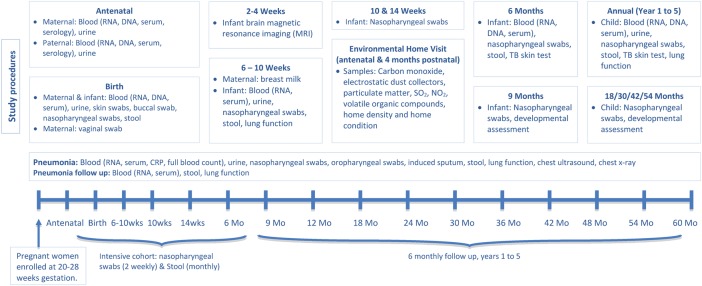
Outline of study visits and samples collected.

The DCHS is a population-based birth cohort study in the Drakenstein area in Paarl, a peri-urban area, 60 km outside Cape Town, South Africa. Pregnant women are enrolled in their second trimester and followed through childbirth; thereafter mother–child pairs are followed until children are at least 5 years old (figure 1). Maternal, paternal and child health are investigated through longitudinal measurements of risk factors in seven areas (environmental, infectious, nutritional, genetic, psychosocial, maternal and immunological) that may impact on child health. Intensive aetiological and risk factor investigations are done during an episode of childhood LRTI.

### Study population

The local community of approximately 200 000 people is of low socio-economic status, live in informal housing or crowded conditions and have high levels of unemployment. Infectious diseases including pneumonia, HIV (antenatal prevalence approximately 30%) and tuberculosis (annual reported incidence 293/100 000) are common. There is a high prevalence of tobacco smoke exposure, alcohol misuse, malnutrition and other poverty-related exposures. Pneumonia is the predominant cause of childhood hospitalisation and death, with the estimated incidence similar to the reported LMIC incidence of 0.22 per child-year in early life.^2^ The population is stable, with little immigration or emigration. More than 90% of the population access healthcare in the public sector including antenatal and child health services. The public health system comprises 23 primary health clinics and one hospital, Paarl Hospital, where all births and hospital care occur. The well-established, free primary healthcare system provides childhood immunisations including 13-valent pneumococcal and *H influenzae* b vaccines as part of the national immunisation schedule.

Consenting pregnant women are enrolled from two primary health clinics serving different populations—TC Newman (serving a mixed race population) and Mbekweni (serving a black African population). Pregnant women who are not enrolled are included in a control cohort; these mother–infant pairs are followed annually to compare outcomes with the active cohort.

### Study procedures and follow-up

Antenatal and postnatal visits are at primary healthcare clinics, while birth, 6-week and annual study visits occur at Paarl hospital (figure 1). Fathers, as identified by mothers, are invited to participate in an antenatal study visit. Infants attend study visits synchronised with the national programme where feasible at 6, 10 and 14 weeks, and 6, 9, 18, 30, 36, 42, 54 and 60 months. Two home visits (antenatally and 4 months postnatally) are done to investigate environmental risk factors.

### Measures

Comprehensive data including biomedical, environmental, psychosocial, demographic, physical and mental health of the mother, father and child and intercurrent morbidity are collected. Specimens (blood, urine, stool, respiratory) are longitudinally taken (figure 1). Urine cotinine, to investigate tobacco smoke exposure, is longitudinally measured. Monitors measuring nitrogen dioxide, sulfur dioxide, carbon monoxide, volatile organic compounds and particulate matter (PM10) exposure over 24 h to 2 weeks are placed in homes; electrostatic dust collectors collect household dust over 2 weeks.

Infant lung function, undertaken for the first time in an African setting, is measured at 6 weeks and annually at Paarl hospital. State-of-the-art measurements in unsedated children during sleep include tidal breathing, exhaled nitric oxide, forced oscillation technique and sulfur hexafluoride multiple breath washout. Lung function is also measured during a LRTI and 4–6 weeks thereafter. Chronic respiratory disease measurements include symptoms, clinical data, lung function and chest X-ray and ultrasound (during an LRTI).

Child neurodevelopmental outcomes are assessed longitudinally with a subsample of infants undergoing brain MRI.

All children have six monthly nasopharyngeal swabs (NPs) and stool specimens collected, while a subset intensive cohort have two weekly NPs and monthly stool samples in the first year. These specimens will enable longitudinal delineation of the child's nasopharyngeal and stool microbiome using targeted (bacterial culture, multiplex real-time PCR for viral and bacterial pathogens) and non-targeted approaches (16srRNA gene sequencing). A similar approach is used for detailed investigation of LRTI aetiology on NP and induced sputum specimens. The maternal microbiome (stool, vaginal, skin, breast milk, NPs) is also studied perinatally (figure 1). The predictive value of the child's microbiome for development of LRTI or chronic respiratory illness is a key area of study.

Specimens from mothers, fathers, children and the environment are processed in a central research laboratory and stored at −80°C, creating a large biobank for future studies.

### Surveillance, community engagement and cohort retention

Measurement of LRTI includes ambulatory and hospitalised pneumonia cases, severe or very severe pneumonia, as defined by WHO criteria. Strong surveillance systems have been established using healthcare workers, cell phones and active surveillance at health facilities. Trained community field workers promote community engagement and enable home visits even in areas where violent crime is common. Several strategies to promote cohort retention are used including automated study visit reminders, a close working relationship with clinical staff, a cell phone system enabling two-way communication with study participants at all times and regular follow-up synchronised with routine visits.

### Ethics

Written informed consent from mothers is renewed annually; informed consent is also obtained from fathers. The study was approved by the Ethics Committee of the Faculty of Health Sciences, University of Cape Town, by Stellenbosch University and the Western Cape Provincial Research committee.

### Sample size

The sample size of 1000 mother-infant pairs is designed to provide at least 550 pneumonia episodes for analyses of LRTI incidence and determinants. We estimate cumulative attrition over 5 years of 20% (including losses due to child mortality) and an expected incidence of LRTI similar to that reported in LMIC.^2^ This sample will provide adequate statistical power to detect relative associations of at least 1.5-fold for prevalent risk factors.

## Conclusion

The DCHS is a unique birth cohort that enables repeated assessments over time to investigate the incidence, aetiology, determinants and long-term impact of early childhood LRTI, on child health in a LMIC. Prenatal recruitment allows assessment of exposures during gestation and after birth, use of a population-based sample helps to eliminate selection biases inherent in case-based approaches and the cohort design ensures clear identification of the time-order of associations. Early, repeated measurement of a broad range of risk factors by a multidisciplinary team and of outcomes with the ability to perform sophisticated measures such as infant lung function, neuroimaging and microbiome investigations are further strengths of the study.

A key focus is the ability to investigate in detail episodes of ambulatory and hospitalised LRTI with state-of-the-art aetiological investigations in the context of high coverage with new conjugate vaccines. Further, the ability to longitudinally measure chronic lung disease presents the opportunity to investigate a key association between childhood communicable and non-communicable diseases. This is a critical area given the importance of chronic respiratory illness as a major non-communicable disease. The study also provides a platform for additional and future studies of child health through the creation of a large, comprehensive biobank.

The study is unique in undertaking longitudinal measurements of child health in the context of a poor African community with a high burden of disease. While most LRTI occurs in LMICs, this is one of the first birth cohort studies in such a setting to investigate these crucial issues using objective, state-of-the-art measures to identify new, improved strategies for prevention and treatment of disease.
